# Overview of Plasma and Platelet Transfusions in Critically Ill Children

**DOI:** 10.3389/fped.2020.601659

**Published:** 2020-11-13

**Authors:** Stacie Kahn, Madhuradhar Chegondi, Marianne E. Nellis, Oliver Karam

**Affiliations:** ^1^Division of Pediatric Critical Care Medicine, NewYork-Presbyterian, Morgan Stanley Children's Hospital, New York, NY, United States; ^2^Division of Pediatric Critical Care Medicine, Stead Family Children's Hospital- Carver College of Medicine, University of Iowa, Iowa, IA, United States; ^3^Pediatric Critical Care Medicine, NewYork-Presbyterian Hospital – Weill Cornell Medicine, New York, NY, United States; ^4^Division of Pediatric Critical Care Medicine, Children's Hospital of Richmond at Virginia Commonwealth University, Richmond, VA, United States

**Keywords:** platelet transfusion, plasma, critical ill children, hemostasis, hemorrhage

## Abstract

Critically ill children are a unique population who frequently receive plasma and platelet transfusions for both active bleeding and mitigation of bleeding risk. While these products are frequently administered, transfusion indications in this population remain unclear, and practice varies across institutions and providers. In this manuscript, we will outline the current evidence regarding plasma and platelet transfusions for hemostasis in the pediatric intensive care setting. For both products, we will describe the product composition, epidemiology, and product indications and discuss the potential risks and benefits involved with the transfusion. We will also discuss knowledge gaps and future areas of research.

## Introduction

Plasma and platelet transfusions are frequently administered to critically ill children to prevent bleeding and help treat active bleeding. Transfusion of whole blood began in the early 1900's, and significant advancements have been made since that time. Plasma was first transfused in 1941 (1), followed by platelet-rich plasma in the 1950's (2) and platelet concentrates in 1965 (3). In 2015, the American Association of Blood Banks (AABB) National Blood Collection and Utilization Surveys noted that 48,000 children received 165,000 apheresis platelet units and 29,000 children received 74,000 plasma units (4, 5). While adult transfusions are subdivided by area of administration, inpatient medicine (including oncology), and critical care being the largest location administrations, this breakdown is notably absent for pediatric patients with the only division being neonatal vs. pediatric. Although these therapies have been used in critically ill children for more than 50 years and based on recent data are still frequent interventions, there are still many unanswered questions regarding optimal transfusion practices.

## Plasma

Plasma transfusions remain a common treatment in medicine, specifically for bleeding in the emergency and intensive care settings. In 2017, it was noted that 3,209,000 units of plasma were distributed to hospitals in the United States with 2,374,000 units being transfused. This remains to be a large amount; however, there is a significant decline of 13% in units transfused since 2015 (6). The most recent practice guidelines around transfusion of plasma from the AABB were released in 2010 (7), whereas the British Society of Hematology released newer guidelines in 2018 (8). Of note, pediatric providers were involved in the creation of the AABB guidelines; however, they are not specific to children. The updated British guidelines also mention children; however, their most recent pediatric specific guidelines were published in 2016 (9). Despite a lack of data in the pediatric population, it is estimated that about 3% of pediatric admissions to the intensive care unit (ICU) received plasma, making this an important area for further research (10).

### Product Composition + Processing

Plasma is the acellular portion of blood and is obtained by centrifugation or apheresis of blood post-donation. While it is ~92% water, the other 8% are vital proteins, such as coagulation factors (factors II, V, VII, VIII, IX, X, XI, and XII; protein C; protein S; antithrombin), albumin, and immunoglobulins (11). Plasma can also be further fractionated and used for its derivatives. Via fractionation, blood banks are able to isolate specific factors, antithrombin III, albumin, immunoglobulins, etc. to be given individually for a variety of indications.

The mean volume of a plasma unit is ~200–250 ml with a small portion of this volume being anticoagulant for storage. Units are stored at −18°C (−0.4°F) or lower as some coagulation factors, specifically factors V and VIII, have shortened half-lives at room temperature (approximately 22°C). The term fresh-frozen plasma (FFP) is used for plasma frozen within 8 h of collection, whereas frozen plasma, also known as FP24 or PF24, is used to refer to plasma frozen after the 8 h mark but within 24 h of collection. Both can be safely stored for up to 1 year post-collection if kept frozen. FFP can then be thawed and kept in the refrigerator for up to 24 h, and some countries allow for FFP or FP to be kept as thawed plasma for up to 5 days (12). The clinical efficacy is similar between FFP and FP, and they are often used interchangeably; however, cryoprecipitate can only be manufactured from FFP (7). For the purposes of this review when we discuss plasma, we will use them interchangeably unless otherwise noted.

Coagulation factor concentrations may vary between single donor units (13). Units can also be pooled from multiple (300–5,000) donors to allow for a more constant concentration of factors (13). In some European countries, plasma is frequently pathogen reduced by one of three methods, the use of methylene blue, amotosalen, or riboflavin, to further decrease the risk of infection. While beneficial for its impact on infectious risk, this process can also significantly impact the amount of coagulation factors present in the unit. The extent to which coagulation factors are impacted depends on the specific pathogen reduction process employed (8, 13). This is not currently standard of practice in the United States. Solvent detergent units, pooled units pathogen reduced by exposure to a solvent and detergent, are the only ones licensed by the Food and Drug Administration (FDA) (7, 13).

### Epidemiology

In addition to a decrease in units transfused over the past several years, there was also an overall decline in plasma collection in the United States by 14%. Approximately 61% of the units collected in 2017 were distributed to centers throughout the country as frozen plasma with 91% manufactured from whole blood collection, and the remaining percentage was collected via apheresis (14). In the United Kingdom, while there was an increase in the use of FFP from 2008 to 2012, there is a notable decline from 2012 onward, with the data extending to 2016 (8).

As noted above, in the US, the number of plasma units transfused decreased by 13% from 2015 to 2017. However, it is notable that the breakdown of product varies from the collection data. FFP and frozen plasma are more evenly split. There was also an increase in the utilization of AB plasma (the universal donor) by 19.8% (14).

### Indications and Dosing

Plasma is given to critically ill children for a variety of reasons with the most common being coagulopathy, bleeding, and prevention of bleeding in those preparing for an invasive procedure. Initiation of extracorporeal membrane oxygenation (ECMO) in critically ill children causes depletion of coagulation factors, fibrinogen depletion, hemolysis, and thrombocytopenia. This results in a need for blood product transfusions, including FFP. Plasma is also used for exchange transfusions for a variety of disorders, which we will not cover in this review. There is a paucity of data in critically ill children regarding the efficacy of plasma transfusions and the risk–benefit ratio; therefore, practices vary widely.

#### Therapeutic Transfusion (Treatment of Bleeding)

Plasma is frequently given to correct coagulopathies in patients with active bleeding; however, there are limited data to support its benefit. In a systematic review, Levy et al. did not find any randomized controlled trials (RCTs) that evaluated the benefit or safety of plasma to treat coagulopathic bleeding in children and adults (15). We looked at critically ill children in 101 pediatric intensive care units (PICUs) in 21 countries and found that 22% of plasma transfusions were given for an indication of critical bleeding. The international normalized ratio (INR) was only significantly improved in patients with a baseline INR of >2.5, supporting that only significant coagulopathy can be improved with plasma transfusion (16). In children and adults with active bleeding who are on vitamin K antagonists for anticoagulation, such as warfarin, FFP transfusions are a common intervention. However, recent adult studies have shown increased incidence of volume overload and ICU admission as a result (17, 18). Refaai et al. compared the use of prothrombin complex concentrates (PCC) with the use of FFP for anticoagulant reversal in adults and found that plasma was independently associated with volume overload (18). In addition, a small single center retrospective study by Sisti et al. found that the use of PCC in pediatric patients with ventricular assist devices, for anticoagulation reversal prior to heart transplant, limited their exposure to exogenous blood products (19). Additional studies are needed to determine the best situation for each product in the pediatric population.

The role of plasma transfusions in pediatric trauma patients with bleeding has been explored. Coagulopathy following trauma has been associated with worse outcomes in the pediatric population (20–22). However, no specific ratio of plasma to other products has been particularly effective in controlling coagulopathy. A recent small retrospective study found no significant difference in the transfusion ratios of surviving and deceased pediatric patients at the 3- or 24-h timepoints (22). In addition, a recent study by Sehdev et al. found no difference in mortality rate of pediatric trauma patients when comparing >2:1 packed red blood cell (pRBC) to plasma ratios and <2:1 pRBC to plasma ratios (23). Therefore, the optimal dose of plasma in children with massive bleeding is still unknown.

#### Prophylactic Transfusion (Prevention of Bleeding)

The use of plasma transfusions in non-bleeding patients is controversial with minimal evidence to support its utility. A study by Deitcher found that in adults with liver disease, an INR of 1.3–1.9 correlated with mean factor levels of 31–65% of factor II, 40–70% of factor V, and 22–60% of factor VII, all adequate concentrations to support hemostasis—further supporting that transfusion would not be indicated for mild coagulopathies (13, 24). Plasma transfusions given prior to invasive procedures have also not been shown to change bleeding rates or outcomes in both children and adults (25–27).

We noted that one-third of patients received plasma transfusions prophylactically and had no bleeding and no planned procedure (16). A meta-analysis including 80 RCTs concluded that there was no evidence of significant benefit for prophylactic plasma transfusion across a range of indications evaluated in both children and adults (28). Likewise, a systematic review in 2013 examining restrictive vs. liberal transfusion of plasma found no RCTs that met their criteria, further highlighting the lack of data surrounding this practice (29). The AABB guidelines for plasma transfusion in children provide the following indications: in massive bleeding, support during treatment of disseminated intravascular coagulation (DIC), as replacement therapy (when specific factor concentrates are unavailable; if indicated during therapeutic plasma exchange), or for emergency reversal of warfarin (7, 30).

#### Dosing

FP transfusions should be ABO compatible but are not required to be identical, and unlike RBC transfusions, AB positive plasma is the universal donor and can be given in emergency situations. The average dose of plasma given is 10–20 ml/kg as this increase factor levels by 15–25% that should allow for hemostasis (31).

### Adverse Effects

There is conflicting evidence regarding the benefits of plasma transfusions for many indications, but the risks of administering plasma are clear. As with all blood products, plasma carries the risk of transfusion reactions, such as transfusion-related acute lung injury, transfusion-associated circulatory overload, and allergic reactions (30). In addition, plasma in the United States is not routinely pathogen reduced, as noted above, and therefore carries the risk of infection. However, transmission of viral infections, such as HIV and hepatitis, from plasma are rare. Children with IgA deficiency are at specific risk for anaphylaxis given the presence of anti-IgA antibodies. Studies in adults have shown associations between plasma transfusions and acute respiratory distress syndrome, nosocomial infections, and septic shock (26, 32). One recent study demonstrated that patients who received FFP developed acute lung injury in 12% of cases, in contrast to 3% in the non-transfused group (24). We performed an observational, prospective single center study in critically ill children and found that 42% of patients who received at least one plasma transfusion in comparison with 8% of patients did not develop new or progressive multiorgan dysfunction syndrome (MODS) (*p* < 0.001), with an adjusted odds ratio of 3.19 (95% CI 1.55–6.58, *p* = 0.002). A significant dose relationship was noted—the more plasma received, the higher the proportion of new or progressive MODS. This study also demonstrated an increased incidence of nosocomial infections, longer PICU length of stay, and a higher 28 day non-adjusted mortality rate in those who received plasma (33).

Plasma transfusions have been shown to have significant risks and impact on clinical outcomes; therefore, given the lack of current evidence to support liberal usage, plasma should not be given prophylactically or used solely as volume expander (7, 9, 30).

### Knowledge Gap and Future Directions

The usage of plasma in both the United States and Europe has decreased in recent years—possibly due to a lack of data regarding indications and appropriate utilization. While there are notably some benefits in patients with major bleeding and severe coagulopathy, the use of plasma in patients with minor coagulation derangements and no bleeding appears to not outweigh the many risks seen in observational studies. RCTs are needed in both the adult and pediatric populations to guide recommendations for plasma transfusion.

## Platelets

Platelets are essential for normal hemostasis. Critically ill children often present with quantitative and/or qualitative platelet abnormalities. The goal of platelet transfusion is to prevent or to limit bleeding. In the 1950's, whole blood and then concentrated platelet transfusions were shown to reduce the mortality associated with bleeding in patients with leukemia. Over the years, there were significant advances in terms of platelet collection, storage, and transfusion practices. However, in children, the indications of platelet transfusions and optimal platelet count thresholds remain unclear.

### Standard Platelet Products

Platelets are commonly prescribed for critically ill children in the PICU (4). Platelet products are pooled from the whole blood derived via a single donor (34).

#### The Whole Blood-Derived Platelet Concentrate (WB-PC)

Also known as pooled platelets or random donor platelets (RDP) are extracted before cooling the blood to <20°C using the platelet-rich plasma or buffy coat method either manually or semiautomated systems. In the US, the platelet-rich plasma is used to extract PC, whereas in Europe, buffy coat is preferred (34). Typically, four to six whole blood units are pooled for a single RDP transfusion. One unit of RDP contains about 55 × 10^9^ platelets suspended in 50–70 ml plasma.

#### The Single Donor PC Using Apheresis Technique (SDA-PC)

Often considered as the gold standard method. Compared with WB-PC, SDA-PC takes a longer time to collect, exposes the donor to an extracorporeal circuit, and involves higher production cost. It contains a higher platelet count, 300 × 10^9^ platelets suspended in 150–300 ml plasma. In addition, apheresis allows the production of specific platelet products, such as human leukocyte antigen (HLA) or human platelet antigen (HPA) compatible (35). One unit of apheresis platelets is equivalent to four to six units of WB-PC. The plasma proteins, including all the coagulation factors, are lower due to platelet added solution.

#### Warm vs. Cold Platelets

Platelets are typically stored at a warmer temperature, 20–24°C under gentle agitation. The advantage of warm storage platelets (WSP) is the longer shelf life of 5–7 days with increased circulation time and beneficial to use prophylactic platelet transfusion in patients at risk for bleeding (36). However, the risk of bacterial contamination is higher with WSP (37). Ever since the renunciation of cold storage platelets (CSP) in the early 1980's, there is an increasing evidence of using CSP in recent years. The CSP are stored at a colder temperature, 1–6°C without agitation. The CSP exhibit better hemostasis, especially useful in patients with active bleeding and major trauma with less risk of bacterial contamination. Though CSP shelf life is shorter than WSP (3 vs. 5 days), the *in vitro* studies suggest extended shelf life for CSP (38). Currently, CSP for the most part are considered experimental, and they are not routinely stocked in blood banks.

### Special Platelet Concentrates

#### Leukocyte-Depleted Platelets

Pre-storage white blood cell (WBC) count reduction is a standard procedure. Leukocytes from the platelet units are depleted using centrifugation and filtration techniques. Leukocytes are depleted following an average of 6 h of contact time to neutralize bacterial contamination risk through the leukocyte mediated phagocytosis (39). The WBC count is <8.3 × 10^5^ in the platelet concentrate. The result of leukocyte depletion is a significant reduction in transmission of bacterial and viral diseases, such as cytomegalovirus (CMV) and Epstein–Bar virus (EBV), and reduced frequency of febrile reactions and transfusion-related immunomodulation (TRIM) (40). Bedside leukocyte depletion is not recommended as it decreases the platelet number in the concentrate.

#### CMV Negative Platelets

Platelets can transmit CMV infection. CMV negative platelets are indicated for CMV negative patients with solid organ or bone marrow transplant, immunodeficiency, and intrauterine transfusion (41).

#### Irradiated Platelets

For all HLA-matched platelet concentrates, pre-transfusion irradiation is a must. Irradiation is also recommended in infants at risk of transfusion-associated graft-vs.-host disease (TA-GVHD) and intrauterine transfusions (42).

#### Pathogen-Reduced Platelets

Multiple techniques are available worldwide to reduce the potential infection risk with platelet transfusion. Pathogen reduction protects against bacterial contamination, and it potentially increases the platelet shelf life up to 7 days (43). In addition, pathogen reduction obviates the need for irradiation. However, these platelets hemostatic efficacy may be lower. Other efficacy and safety issues of pathogen-reduced platelets are currently under investigation (43).

#### Cryopreserved Platelets

These platelets are considered an experimental product, and due to storage at −80°C, they require standardized thawing protocols, which may not be possible at all centers. The advantage of cryopreserved platelets is a significant increase in shelf life, up to 2 years. However, the efficacy and safety issues are not well-described (44).

### Epidemiology of Platelet Transfusion

Over 2.2 million platelet units are transfused annually in the US (45), and 275,000 units in UK (46). The current trend shows a decline in the surplus inventory (45, 46). In 2015, the AABB reported 165,000 apheresis platelet units among 48,000 children in the US (5). The rate of platelet transfusion in critically ill children is 3.4% during their PICU stay (4). Over two-thirds of platelet transfusions are prescribed prophylactically and remaining for active bleeding (4). The existing literature is limited in children. The platelet transfusion practice follows adult transfusion protocols, and there is a wide variation in platelet transfusion practice across US health care centers and globally (4, 47).

Thrombocytopenia is defined as a platelet count of <150 × 10^9^/L. In critically ill children at the time of PICU admission, the reported incidence of thrombocytopenia was 17%, and during the PICU stay, 25% of children were thrombocytopenic (48). The underlying mechanisms of thrombocytopenia are decreased production, increased destruction of platelets, and dilutional. Among the critically ill children, common etiologies for thrombocytopenia may include sepsis, chemotherapy, DIC, MODS, and hemolytic uremic syndrome. Heparin-induced thrombocytopenia (HIT), hemophagocytic lymphohistiocytosis (HLH), and massive transfusion are less common etiologies (49, 50). Qualitative platelet defects are more common due to sepsis, exposure to hypothermia, cardiopulmonary bypass, chemotherapy, antiplatelet drugs, and rarely due to hereditary disorders (47). This platelet dysfunction might also be another factor in prescribing transfusions at higher platelet count.

### Indications for Platelet Transfusion

In critically ill children, platelets are transfused as prophylactic or as therapeutic for the ongoing bleeding. Since the transient rise of platelet count in these children does not address the underlying etiology, the main aim of transfusion is to prevent major bleeding. Thrombocytopenia has been identified as an independent risk factor for major bleeding and mortality in critically ill children at admission or during their stay in the PICU (48, 51, 52). In children with a higher risk of mortality, platelet transfusion may be beneficial, whereas in children with low risk of mortality, the transfusion risks may outweigh the benefits (52). This relationship suggests that besides thrombocytopenia, disease severity should be considered while prescribing platelet transfusion. Platelet transfusions are also indicated for reversal of antiplatelet drugs, such as aspirin and clopidogrel; however, clear guidelines are lacking.

#### Therapeutic Platelet Transfusion

The therapeutic transfusion of platelets is indicated for clinically significant bleeding associated with thrombocytopenia or due to dysfunctional platelets. With major bleeding, often, platelets are transfused to keep platelet count >50 × 10^9^/L and, while on ECMO support, above 100 × 10^9^/L (53). However, without a clear definition of major bleeding and a paucity of data to guide therapeutic platelet transfusions in critically ill children, indications for transfusion are unclear. One cannot apply the existing adult platelet transfusion guidelines in children, which are based on expertise opinions (54).

The current practice in children with massive bleeding is to give empiric transfusion of WB-derived platelets and RBCs transfused between 1:1 and 1:2 ratio until the bleeding resolves. When using apheresis platelets, the ratio is between 1:5 and 1:10, as each unit is concentrated. However, this data is based on RCTs in adult patients (55).

While therapeutic transfusion strategy may result in a lower rate of platelet transfusions, reduced cost, and resource utilization, the patient safety and risk of major bleeding are of concern. There is conflicting evidence as to the efficacy of platelet transfusions preventing bleeding. Though adult studies reported no relationship between the lowest platelet count and the risk of bleeding in adult oncology patients (56), in a prospective observational study, Moorehead et al. reported an increased risk of major bleeding with low platelet count in critically ill children (51). Most recently, a large RCT in pre-mature neonates showed increased bleeding and/or mortality in those transfused at a liberal threshold (50 × 10^9^ cells/L) as compared with a restrictive threshold (25 × 10^9^ cells/L) (57). Animal models have suggested that it is not thrombocytopenia alone that leads to bleeding, but rather thrombocytopenia in the setting of inflammation (58).

Platelet transfusions have been associated with increased morbidity and mortality in children (49, 52). In an international point prevalence study, we showed an independent association between dose of platelets and mortality (4). A recent study by Cashen et al. suggested that platelet transfusions are associated with increased risk of mortality, bleeding, and thrombosis in children receiving ECMO support (59).

#### Prophylactic Platelet Transfusion

The majority of platelet transfusions in children are given prophylactically to prevent bleeding (4). Currently, there is a lack of evidence to suggest a platelet count threshold that can be generalizable to all children. Previous studies report that between 50 and 67% of platelet transfusions are prescribed to prevent bleeding in children, and that the median pre-transfusion platelet count was between 39 and 50 × 10^9^/L (4, 52). In a recent point prevalence study, we reported that 34% of children received platelet transfusion even when the platelet count was above 50 × 10^9^/L to meet the arbitrary platelet count threshold (4). The current transfusion threshold recommended by the AABB with hypoproliferative thrombocytopenia is 10 × 10^9^/L. This threshold platelet count is based on the Platelet Transfusion Trigger Trial, which reported no significant difference in the occurrence of bleeding comparing the platelet count 10 × 10^9^/L vs. 20 × 10^9^/L in adolescents and adults with acute myeloid leukemia (AML) undergoing induction therapy (60). A secondary analysis of the prophylactic platelet dose (PLADO) trial in adults and children with hematological malignancies reported a poor association with the thrombocytopenia severity and risk of bleeding (61). Studies in neonates also suggest that the risk of clinically significant bleeding including intraventricular hemorrhage is likely not related to the degree of thrombocytopenia (62, 63). The poor correlation between thrombocytopenia and risk of bleeding suggests considering other factors, such as underlying etiology, to assess the bleeding risk.

There is a wide variation in the practice of prophylactic platelet transfusion in children, and most of the clinical practice guidelines are based on expert opinion. Often the platelet count threshold for transfusion varied depending on the geographic location of clinical practice (29), with underlying clinical indication and diagnosis (4, 5, 64, 65). The clinical practice of platelet transfusion for hypoproliferative thrombocytopenia cannot be applicable in thrombocytopenia due to immune-mediated platelet destruction. The American Society of Hematology recommends platelet transfusion only with life-threatening bleeding or during the preparation for surgical procedures (66). In critically ill children with qualitative platelet dysfunction, often platelets were transfused even with normal platelet count. Currently, there is no consensus for transfusion threshold platelet count (47). The AABB, in adults, recommend prophylactic platelet transfusion at the following threshold platelet counts: for hospitalized patients with hypoproliferative thrombocytopenia at a threshold platelet count of 10 × 10^9^/L, for patients requiring elective central venous catheter placement at 20 × 10^9^/L, patients with a lumbar puncture at 50 × 10^9^/L, and patients with major non-neuraxial surgery at 50 × 10^9^/L (67).

#### Dosing

Unlike adults, the platelet doses are smaller in children. However, the platelet type and preparation methods are similar. The standard dose for platelet transfusion in adults is typically 3 × 10^11^−6 × 10^11^ platelets. The PLADO trial compared the prophylactic platelet transfusion at low (1.1 × 10^11^), medium (2.2 × 10^11^), and high (4.4 × 10^11^) doses of platelets per square meter (m^2^) of body surface area (BSA) among adults and children with hematologic cancers and solid tumors undergoing hematopoietic transplant and chemotherapy. This study reported no difference with the bleeding incidence among patients who received low or high dose platelet transfusion (68). The current clinical practice suggests 1 unit of pooled platelets for every 10 kg body weight and 5–10 ml/kg of apheresis platelets. Expert guidelines recommend not to transfuse more than six pooled or a single apheresis unit at a time.

### How to Transfuse Platelets

The volume of the WB-PC is 50 ml, whereas the apheresis unit volume ranges from 200 to 300 ml. Up to 95%, this volume is plasma or plasma and platelet additive solution (PAS). Volume reduced platelet units are only recommended when circulatory overload risk is significant. Besides delaying the platelet unit release from the blood bank, the volume reduction process decreases platelet count by 20% and activates the platelets (42). Platelets must be used within 4 h once they are released from the blood bank and complete transfusion within 30 min. While transfusing platelets, a filter with 80–170 micropores must be used to remove the aggregates. The benefit of ABO matching platelet transfusion is inconclusive, and currently, there is no consensus guideline that exists (69). However, Rh compatibility is highly desirable in childbearing age women to prevent Rh alloimmunization as platelet units invariably contain few RBCs. If the platelet transfusion is Rh incompatible, an anti-D immunoglobulin should be administered within 48 h (70). Withholding immunoprophylaxis for Rh incompatible platelet transfusion is safe, especially in males and women past the reproductive age as the rate of anti-D alloimmunization is very low with increased SDA-PC utilization (71).

### Platelet Dose Response

Response to the platelet count is measured by the count increment (CI), which is defined as the platelet count increase within 1 h post-transfusion. With adequate platelet dose transfusion, the platelet CI should be above 20% of the pre-transfusion platelet count if measured within 10–60 min and higher than 10% if measured with 24 h post-transfusion. By body weight, one unit of platelet transfusion per 10 kg should increase platelet count by 35,000–50,000/mm^3^ and by 7,000–11,000/mm^3^/m^2^ of BSA. In neonates and infants, a dose of platelet volume 5–10 ml/kg should increase the platelet count by 50,000–100,000/mm^3^ (70). Though the CI method is simple, it has been shown not to be very accurate. Another accurate method to assess post-transfusion response is corrected count increment (CCI), which includes BSA and the number of platelets transfused. The CCI is usually measured within 10–60 min post-transfusion, and its value is expected to be >7,500/μl × 10^11^ platelets transfused/m^2^ BSA. Lack of adequate platelet CI response suggests platelet refractoriness, and it is defined as CCI <7,500 with two subsequent platelet transfusions. The platelet refractoriness is often seen with increased platelet consumption due to DIC, acquired HLH, splenomegaly, and medications, such as amphotericin-B therapy. Managing the underlying clinical condition improves platelet refractoriness. Failure to have the CCI response within 1 h of post-transfusion in the absence of hypoproliferative and consumptive pathology suggests immune-mediated causes, such as presence of HLA or HPA antibodies (70). Immune-mediated platelet refractoriness conditions require HLA matching, cross-matching, and antibody specificity prediction methods to identify compatible platelet units. If antibody-matched platelets are not available, in the absence of bleeding, rituximab, recombinant factor VIIa, plasma exchange, intravenous immunoglobulins, and platelet continuous infusion can be used (68).

### Platelet Transfusion Reactions

Common transfusion reactions associated with platelet transfusion are also similar to other blood component transfusions. Since the platelet storage temperature is around 24°C, almost all bacteria can grow, and therefore platelet concentrates have the highest risk of bacterial contamination as compared with other blood components. Compared with WB-PC, the risk of bacterial infection is five times with the SDA-PC (34). Febrile non-hemolytic transfusion reactions (FNHTR) and transfusion-related acute lung injury (TRALI) are common with platelets compared with other blood product transfusions (34). Other risks include alloimmunization with HLA, HPA, and Rh antigens and graft-vs.-host disease (34). A recent study reported an increased risk of organ dysfunction, sepsis, nosocomial infections, prolonged ICU stay, and mortality with platelet transfusions (49). The use of PAS reduces the plasma content of the platelets by two-thirds and decreases the incidence of adverse reactions related to plasma (34).

### Knowledge Gap and Future Directions

There had been a significant advancement in terms of platelet preparation, storage, and transfusion safety. There remain opportunities to improve platelet transfusion practice in children. Using the WB-PC and SDA-PC is standard practice. However, the superiority of these products in terms of dose response and overall safety is still under investigation. A recent interest in using cold platelet transfusion in children needs further evaluation. The relevance of ABO compatibility in platelet transfusion is still controversial. The current prophylactic transfusion practice in the PICU is based on expert opinion, and there is a lack of objective evidence to favor prophylactic vs. therapeutic platelet transfusion. The optimum platelet count threshold for transfusion is significantly variable depending on the underlying etiology, clinical practice setting, and geographic location, which needs further evaluation (72). At present, there is a lack of evidence to replace platelet transfusions with the newer platelet substitutes, such as artificial platelets, recombinant interleukin-6 or-11, and thrombopoietin mimetics (73).

## Conclusion

Over the last century, significant advancements have been made in the field of transfusion medicine. Platelet and plasma transfusions have become safer in many ways with the changes in processing methods and advances in storage techniques. While these products are commonly used in critically ill children, there is still much to be learned regarding the optimal usage of platelets and plasma in this group.

## Author Contributions

All authors listed have made a substantial, direct and intellectual contribution to the work, and approved it for publication.

## Conflict of Interest

The authors declare that the research was conducted in the absence of any commercial or financial relationships that could be construed as a potential conflict of interest.
